# Retrograde TrkAIII transport from ERGIC to ER: a re-localisation mechanism for oncogenic activity

**DOI:** 10.18632/oncotarget.5802

**Published:** 2015-09-22

**Authors:** Antonietta Rosella Farina, Lucia Cappabianca, Pierdomenico Ruggeri, Luciana Gneo, Rita Maccarone, Andrew Reay Mackay

**Affiliations:** ^1^ Department of Applied Clinical and Biotechnological Sciences, University of L'Aquila, L'Aquila, Italy; ^2^ Department of Medical-Surgical Science and Biotechnology, University of Rome “La Sapienza”, Latina, Italy

**Keywords:** TrkAIII oncoprotein, retrograde transport, activation mechanism, neuroblastoma, endoplasmic reticulum intermediate compartment

## Abstract

In human SH-SY5Y neuroblastoma (NB) cells, nascent immature N-glycosylated 110kDa TrkA moves rapidly from the endoplasmic reticulum (ER) to the Golgi Network (GN), where it matures into the 140kDa receptor prior to being transported to the cell surface, creating GN and cell surface pools of inactive receptor maintained below the spontaneous activation threshold by a full compliment of inhibitory domains and endogenous PTPases. In contrast, the oncogenic alternative TrkAIII splice variant is not expressed at the cell surface but re-localises to intracellular membranes, within which it exhibits spontaneous ERGIC/COPI-associated activation and oncogenic Akt signalling. In this study, we characterise the mechanism responsible for TrkAIII re-localisation. Spontaneous TrkAIII activation, facilitated by D4 IG-like domain and N-glycosylation site omission, increases spontaneous activation potential by altering intracellular trafficking, inhibiting cell surface expression and eliminating an important inhibitory domain. TrkAIII, spontaneously activated within the permissive ERGIC/COPI compartment, rather than moving in an anterograde direction to the GN exhibits retrograde transport back to the ER, where it is inactivated. This sets-up self-perpetuating TrkAIII re-cycling between the ERGIC and ER, that ensures continual accumulation above the spontaneous activation threshold of the ERGIC/COPI compartment. This is reversed by TrkA tyrosine kinase inhibitors, which promote anterograde transport of inactivated TrkAIII to the GN, resulting in GN-associated TrkAIII maturation to a 120kDa species that is degraded at the proteasome.

## INTRODUCTION

The developmental and stress-regulated alternative TrkAIII splice variant of the NGF receptor tropomyosin-related kinase, TrkA, is expressed by advanced stage human neuroblastomas (NBs), associates with poor prognosis and post therapeutic relapse in unfavourable high TrkA expressing tumours and exhibits oncogenic activity in NB models [1-6]. TrkAIII oncogenic activity is caused by exon 6/7 skipping, which results in the omission of the extracellular D4 IG-like domain and several N-glycosylation sites, important for optimising intracellular trafficking, correct cell surface localisation, ligand binding and preventing spontaneous ligand-independent receptor activation [1, 7-9]. As a consequence and in contrast to TrkA, TrkAIII is not expressed at the cell surface but re-localises to intracellular membranes, within which it exhibits spontaneous ligand-independent activation, leading to oncogenic signalling through Akt but not Ras from this alternative location. Signalling from TrkAIII differs to classical signalling from cell surface ligand-activated TrkA through Ras/MAPK, suggesting that the re-localisation of TrkAIII is fundamental for it's oncogenic activity [1]. This hypothesis is supported by recent reports that the oncogenic activity of several receptor tyrosine kinase (RTK) oncogenes depends upon their re-localisation [10]. Mutation-activated FLT3 and c-KIT oncogenes, important in haematological malignancies, are retained within the ER, exhibit ER-associated spontaneous activation and oncogenic signalling through Akt/STAT5/PIM [11-12]. The Trk-T3 oncogene, involved in thyroid cancer, re-localises to ER exit sites (ERES), exhibits spontaneous activation within ERES-associated COPII vesicles and induces oncogenic signalling from this alternative location through Akt but not Ras, similar to that reported for the TrkAIII oncoprotein [1, 13].

The re-localisation of FLT3-ITD and c-Kit-ITD to the ER results from duplication of the internal tandem juxta-membrane domains, which impairs ER exit and facilitates spontaneous activation by compromising an important receptor inhibitory domain [11, 12]. The re-localisation of Trk-T3 to the ERES/COPII compartment results from TrkA extracellular domain substitution with a chimeric Trk-fused gene (TFG) sequences that regulate COPII vesicle formation at ERES, with the elimination of important TrkA extracellular domain sequences further facilitating spontaneous activation [13]. Whatever mechanisms result in RTK oncogene re-localisation, all induce oncogenic signalling from an alternative substrate context [1, 10-13].

Interestingly, the mere overexpression of TrkA in some cell types results in spontaneous intracellular activation and altered signalling. In human SV40-immortalised 293 kidney cells, TrkA overexpression results in spontaneous activation within the ERGIC, which causes GN disruption and inhibits further GN-associated TrkA maturation. This augments intracellular accumulation, facilitates spontaneous activation and results in altered signalling through Akt but not Ras from this alternative site, also similar to that reported for the TrkAIII oncoprotein [1, 14]. This cell-specific effect most likely reflects slower rates of intracellular TrkA trafficking, maturation and cell surface translocation, leading to aberrant accumulation and spontaneous activation within the ERGIC, perpetuated by GN disruption [14].

In pursuit of novel therapeutic strategies to inhibit RTK oncogenic activity, therefore, it is not only important to characterise the mechanisms responsible for oncogenic RTK activation and the oncogenic signalling pathways activated but also to identify the specific sites to which RTK oncogenes re-localise, and the mechanisms that cause re-localization and facilitate spontaneous activation. In the present study, we report a novel mechanism to explain the re-localisation of the TrkAIII oncoprotein in human NB cells. This mechanism is characterised by activation-dependent retrograde TrkAIII recycling from the ERGIC back to the ER, which reduces anterograde TrkAIII transport to the GN, impedes further GN-associated TrkAIII maturation and sets-up self-perpetuating TrkAIII accumulation above the threshold for spontaneous activation within the ERGIC/COPI compartment, resulting in oncogenic Akt signalling from this alternative location. This mechanism is prevented by TrkA inhibitors, which promote anterograde trafficking of inactivated TrkAIII from the ERGIC to the GN, resulting in GN-associated maturation of TrkAIII to a novel 120kDa species that fails to reach the cell surface and is degraded at the proteasome.

## RESULTS

### TrkA localises to two distinct pools of inactive receptors in SH-SY5Y cells

**Figure 1:**
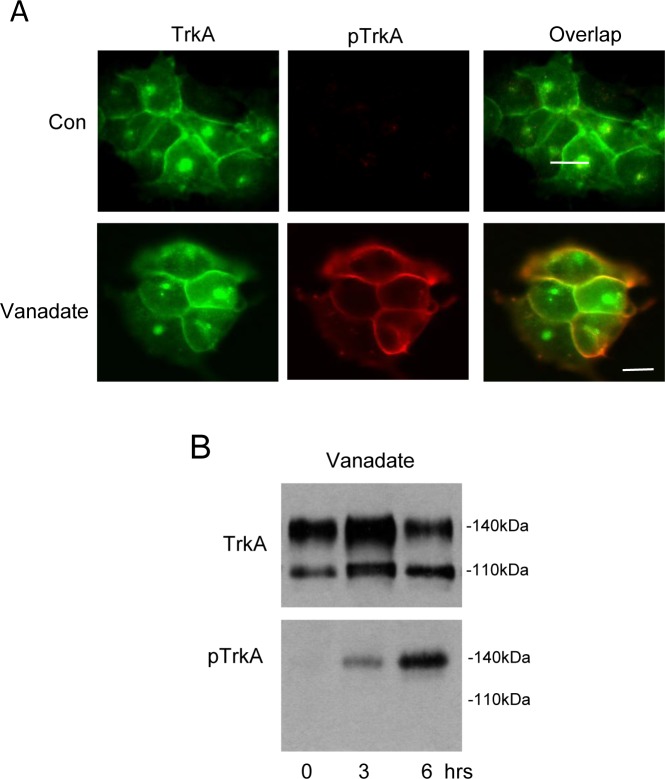
Cell surface but not GN-associated TrkA is activated by Sodium Orthovanadate **A.** Indirect IF demonstrating overlap (yellow, bottom right panel) in surface and GN-associated TrkA (green, top and bottom left panels) and phosphorylated TrkA (pTrkA: red, top and bottom middle panels) in untreated (Con) and sodium orthovanadate-treated (Vanadate; 0.1mM for 3 hours) TrkA SH-SY5Y cells (bars = 10μm). **B.** Western blot demonstrating the effect of sodium orthovanadate (vanadate; 0.1mM for 3 and 6 hours) on total (TrkA) and tyrosine phosphorylated TrkA (pTrkA) levels.

We have previously reported [3] and confirm here (Figure 1A) that TrkA trafficking from ER to GN and then to the cell surface in SH-SY5Y NB cells occurs without spontaneous activation and results in two distinct steady-state pools of inactive TrkA receptors at the cell surface and within the GN, the latter overlapping closely with the GN marker GM130 [3]. Treatment of TrkA SH-SY5Y cells with the PTPase inhibitor sodium orthovanadate (Vanadate, 0.1mM for 3 hours) induced tyrosine phosphorylation of cell surface but not GN-associated 140kDa TrkA, implicating endogenous PTPases in preventing spontaneous activation of cell surface 140kDa TrkA but not GN-associated TrkA (Figure 1A, 3 hours treatment and Figure 1B, 3 and 6 hours treatment).

### CEP-701 alters the localisation of intracellular immature N-glycosylated 100kDa TrkAIII

**Figure 2:**
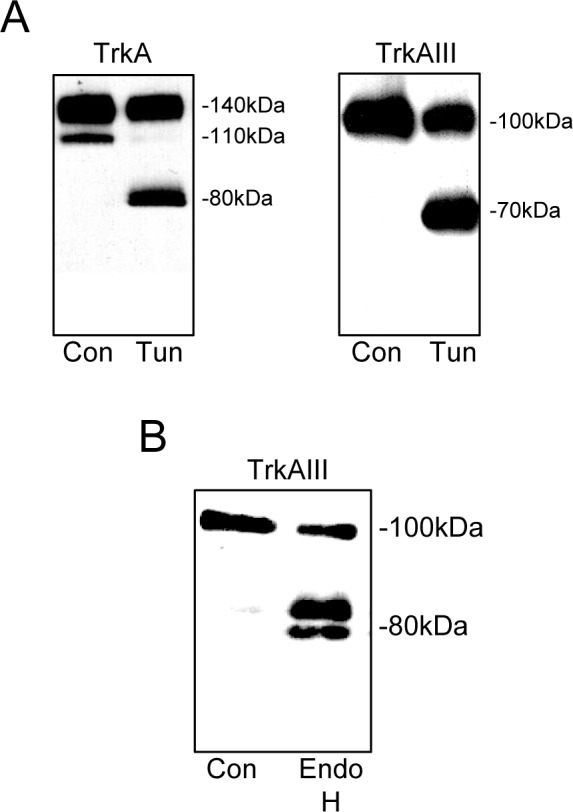
100kDa TrkAIII is an immature N-glycosylated receptor Western blots demonstrating the effect of tunicamycin (Tun, 0.1mM for 16 hours) on the molecular size of A) TrkA and TrkAIII, in total TrkA SH-SY5Y and TrkAIII SH-SY5Y cell extracts (30μg). B) Western blot demonstrating degradation of immunoprecipitated 100kDa TrkAIII (Con) by Endoglycosidase H (Endo H).

The N-glycosylation status of 100kDa TrkAIII was compared to that of 110kDa and 140kDa TrkA following overnight treatment with the N-glycosylation inhibitor tunicamycin (5μg/ml) [15]. Tunicamycin induced expression of 80kDa TrkA and 70kDa TrkAIII core proteins (Figure 2A), confirming an N-glycosylated status for 100kDa TrkAIII, 110kDa and 140kDa TrkA proteins (Figure 2A). Incubation of 100kDa TrkAIII immunoprecipitates with endoglycosidase H (250U per reaction) resulted in TrkAIII degradation to 80-85kDa fragments, confirming an immature N-glycan status for 100kDa TrkAIII (Figure 2B) [16].

**Figure 3:**
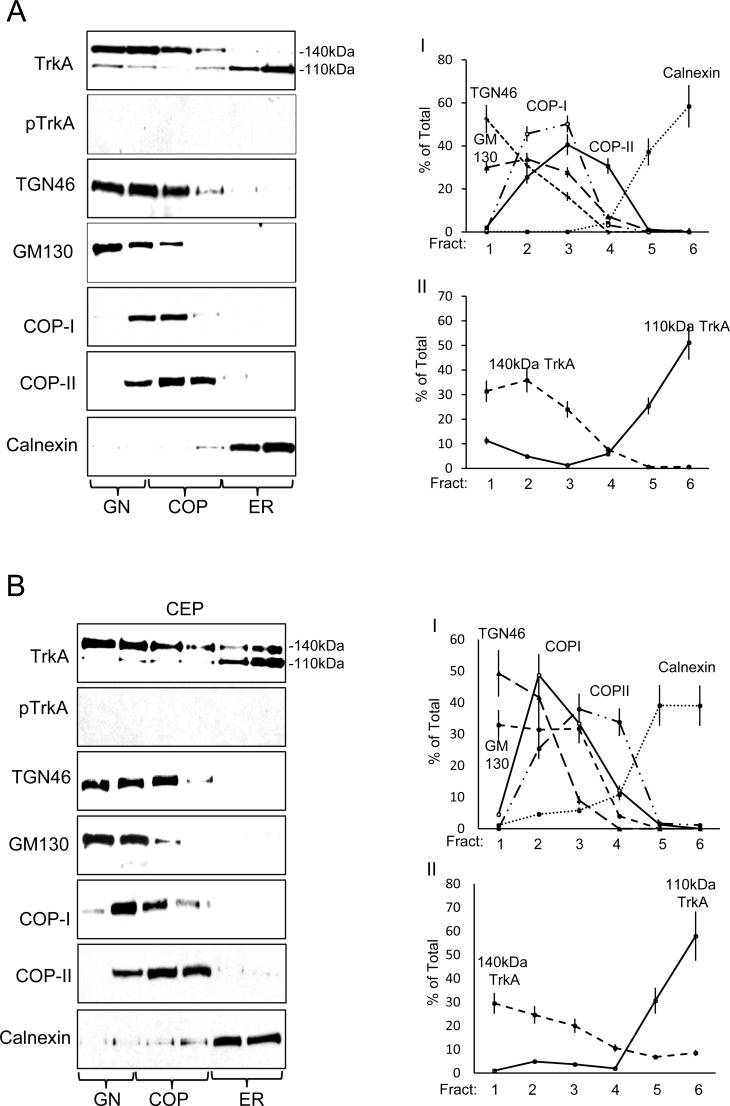

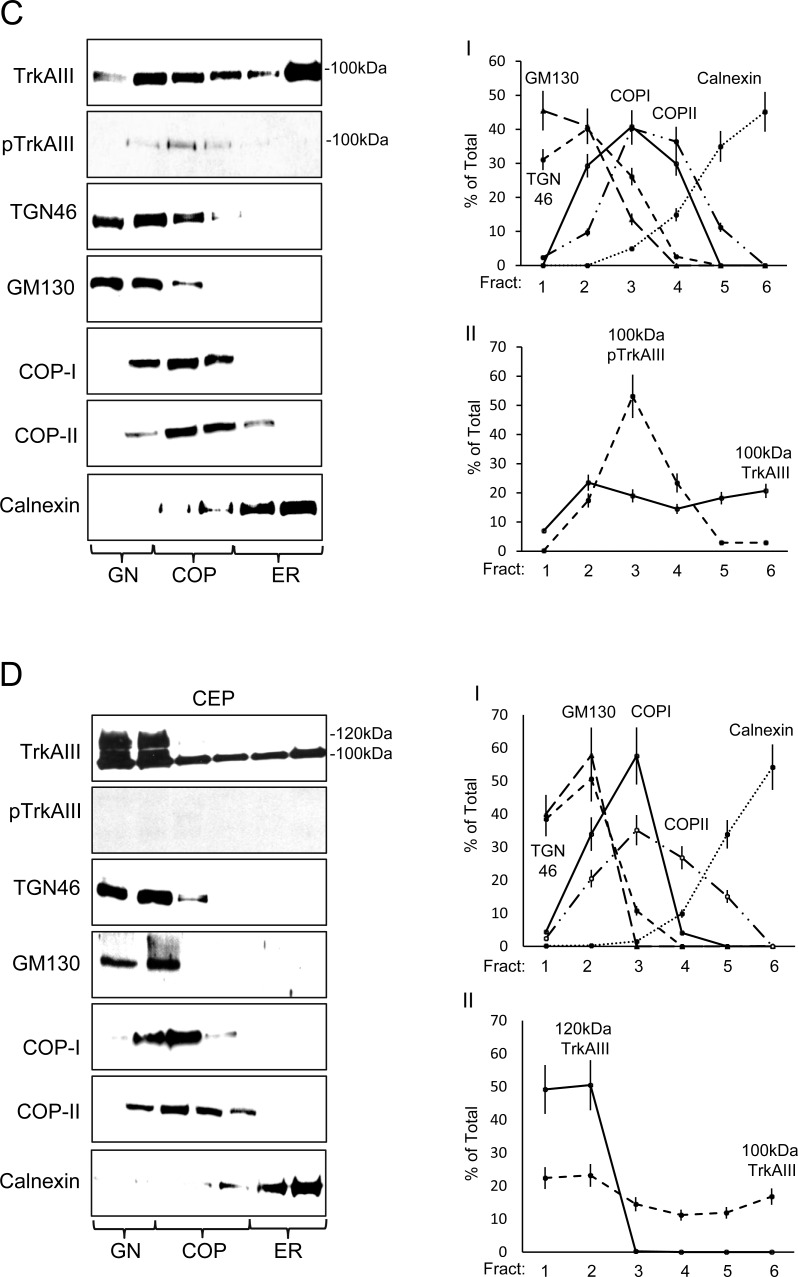
TrkAIII differs to TrkA in steady-state intracellular distribution and activation Representative Western blots plus line graphs demonstrating steady state levels of: 140kDa and 110kDa TrkA; Y674/675 phosphorylated TrkA (pTrkA); 120kDa and 100kDa TrkAIII; Y674/675 phosphorylated TrkAIII (pTrkAIII); TGN46; GM130; COP-I, COP-II and calnexin in density-gradient ultra-centrifugation purified intracellular membranes fractions from: **A.** untreated TrkA SH-SY5Y cells; **B.** CEP-701-treated (CEP; 100nM for 12 hours) TrkA SH-SY5Y cells; **C.** untreated TrkAIII SH-SY5Y cells; and **D.** CEP-701-treated (CEP; 100nM for 12 hours) TrkAIII SH-SY5Y cells. Line graphs depict GM130, TGN46, COP-I, COP-II and calnexin levels in: AI) untreated TrkA SH-SY5Y cells; BI) CEP-701-treated (CEP; 100nM for 12 hours) TrkA SH-SY5Y cells; CI) untreated TrkAIII SH-SY5Y cells; and DI) CEP-701-treated (CEP; 100nM for 12 hours) TrkAIII SH-SY5Y cells. Levels of 110kDa and 140kDa TrkA are displayed in AII) for untreated TrkA SH-SY5Y cells and in BII) for CEP-701-treated TrkA SH-SY5Y cells (CEP). Total and tyrosine phosphorylated 100kDa TrkAIII levels are displayed in CII) for untreated TrkAIII SH-SY5Y cells and levels of 100kDa and 120kDa TrkAIII displayed in DII) for CEP-701-treated TrkAIII SH-SY5Y cells (CEP) (presented as mean ± s.d. % total, from densitometric analysis of three independent experiments performed in duplicate).

The steady state intracellular distribution of TrkA and TrkAIII was compared in density-gradient ultracentrifugation purified membrane fraction prepared under untreated (control) and CEP-701-treated (100nM for 16 hours) conditions (Figure 3). In TrkA SH-SY5Y cells, 76.6+6.9% of immature 110kDa TrkA was ER-associated (calnexin positive fractions 5 and 6), 7.2+0.9% COP-associated (COPI/II positive fractions 3 and 4) and 16.2+1.5% GN-associated (TG46/GM130 positive fractions 1 and 2) (Figure 3A I-II), whereas 1.2+0.1% of mature 140kDa TrkA was ER-associated (fractions 5 and 6), 34.9+2.4% COP-associated (fractions 3 and 4) and 63.8+3.8% GN-associated (fractions 1 and 2) (Figure 3A I-II), confirming GN-associated 140kDa TrkA maturation. CEP-701 (100nM) did not significantly alter the steady state distribution of either 110kDa or 140kDa TrkA (Figure 3B I-II). TrkA tyrosine phosphorylation was not detected (Figure 3A).

The steady state distribution of immature 100kDa TrkAIII differed to that of immature TrkA and was 38.9+3.3% ER-associated (fractions 5 and 6), 33.5+2.8% COP-associated (fractions 3 and 4), 7+8.3% associated with GN-COP-negative fraction 1 and 23.5+2.7% associated with GN-COP-positive fraction 2 (Figure 3C I-II), indicating that immature 100kDa TrkAIII does not accumulate in COP-negative, TGN46/GM130 positive GN membranes. Spontaneous TrkAIII Tyrosine phosphorylation was detected primarily in COPI/II positive membranes (76.5+7% in COP positive fractions 3 and 4), whereas only 5.8+0.5% was ER-associated (fractions 5 and 6) and 17.5+2.1% GN-associated (fractions 1 and 2) (Figure 3C I-II). No other TrkAIII species were detected.

CEP-701 inhibited TrkAIII tyrosine phosphorylation and significantly increased localization of 100kDa TrkAIII to 45.6±4.5% in GN membranes (fractions 1 and 2; *versus* untreated controls *P* = 0.019, df = 6) and significantly reduced TrkAIII in COP-membranes to 25.7±2.6% (fractions 3 and 4; *versus* untreated controls P = 0.0065, df = 6) and in ER-membranes to 28.7±2.9% (fractions 5 and 6 *versus* untreated controls *P* = 0.0035, df = 6), indicating increased transport to the GN (Figure 3D I-II). CEP-701 also induced the expression of an additional 120kDa TrkAIII species, localized 51±7.6% to COP negative TGN46/GM130 positive GN membrane fraction 1 and 49±7.4% to TGN46/GM130 positive-COP positive fraction 2 (Figure 3D I-II). As a consequence, total TrkAIII (100kDa plus 120kDa) levels were significantly increased to 34±1.7% (*versus* untreated controls *P* < 0.0001, df = 6) in COP negative TGN46/GM130 positive GN fraction 1, to 33.7±5% in TGN46/GM130 positive COP positive membrane fraction 2 (*versus* untreated controls *P* = 0.0013, df = 6) and significantly reduced to 15.6±1.7% in COP-membranes (fractions 3 and 4; *versus* untreated controls *P* < 0.0001, *n* = 6) and 16.7±1.8% (fractions 5 and 6; *versus* untreated controls *P* < 0.0001, df = 6) in ER-membranes (Figure 3D I-II). Therefore, CEP-701 promotes TrkAIII transport from ER to GN and induces GN-associated 120kDa TrkAIII maturation (Figure 3D).

### Activated TrkAIII recycles back to the ER

Metabolically labelling was used to further interrogate TrkA and TrkAIII trafficking over 60 minutes in density gradient ultracentrifugation separated membranes.

**Figure 4:**
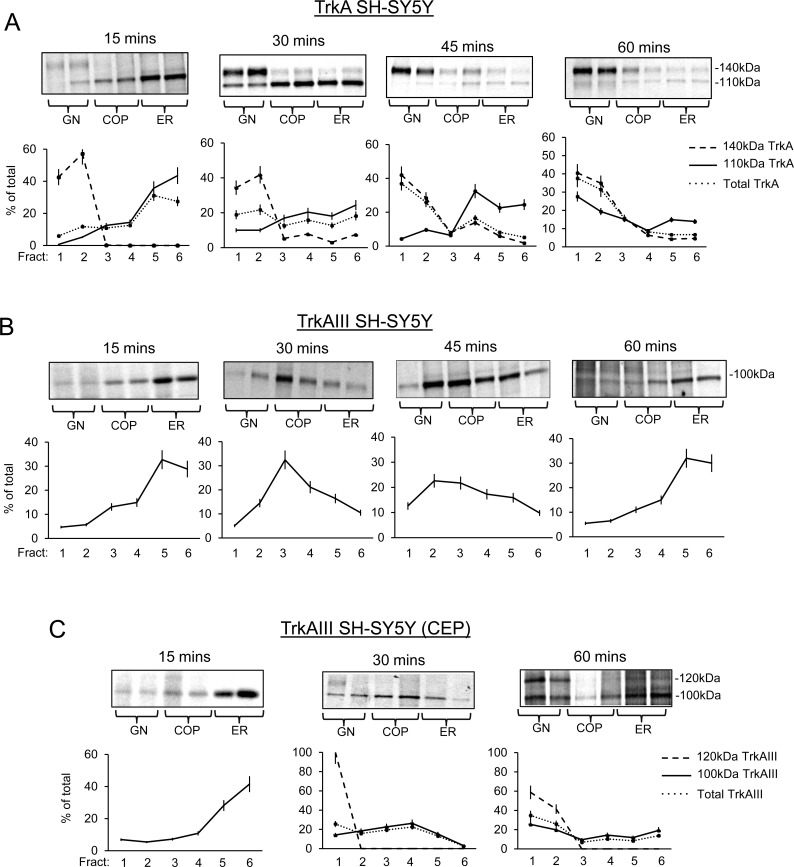
TrkAIII but not TrkA returns to the ER Representative autoradiographs plus line graphs demonstrating time-dependent changes in the distribution of pulse labelled 110kDa/140kDa TrkA and 100kDa/120kDa TrkAIII in density-gradient ultra-centrifugation purified GN, COP vesicle and ER membrane fractions from: **A.** untreated TrkA SH-SY5Y cells; **B.** untreated TrkAIII SH-SY5Y cells; and **C**. TrkAIII SH-SY5Y cells treated with 100nM CEP-701 (CEP), at the times indicated in minutes, following metabolic pulse labelling (presented as the mean ± s.d. percentage of total, derived from densitometric analysis of autoradiographs from independent experiments, performed in duplicate).

In TrkA SH-SY5Y cells (Figure 4A) at 15 minutes post-labelling, 79.2±10.8% of immature 110kDa TrkA was ER-associated (fractions 5 and 6), 26.9±2.3% COP-associated (fractions 3 and 4), 5.9±0.6% GN-associated (fractions 1 and 2) and low-level 140kDa TrkA was detected exclusively in GN fractions 1 and 2. Total TrkA (110kDa plus 140kDa) was, therefore, 58.7±4.6% ER-associated, 23.5±1.8% COP-associated and 17.8±1.5% GN-associated. By 30 minutes, ER-associated 110kDa TrkA had reduced to 42.5±3.6% (fractions 5 and 6; *P* = 0.0007, *n* = 6), COP-associated 110kDa TrkA had increased to 37.3±3.2% (fractions 3 and 4; *P* = 0.002, df = 6), GN-associated 110kDa TrkA had increased to 20±1.7% (fractions 1 and 2; *P* < 0.0001, df = 6) and 140kDa TrkA was more clearly detected in GN membranes (fractions 1 and 2). Total TrkA (110kDa plus 140kDa) was, therefore, reduced to 30.9±3.1% in ER membranes (fractions 5 and 6; *P* = 0.0001, df = 6) and increased to 40.6±4% in GN membranes (fractions 1 and 2; *P* = 0.0001, df = 6). This change continued through 45 minutes and by 60 minutes resulted in a further reduction in ER-associated immature 110kDa TrkA to 28.8+2% (fractions 5 and 6, *versus* 15 minutes *P* < 0.0001, df = 6); 23.8±1.7% in COP-membranes (fractions 3 and 4) and a significant increase to 46.9± 3.4% in GN membranes (fractions 1 and 2; verses 15 minutes *P* < 0.0001, df = 6). This was associated with further increases, at both 45 and 60 minutes, in GN-associated (fractions 1 and 2) 140kDa TrkA levels. At 60 minutes, total TrkA (110kDa plus 140kDa) levels had reduced to 13.4±1.2% in ER membranes (fractions 5 and 6; *versus* 15 minutes *P* < 0.0001, *n* = 6) and increased to 69±6.4% in GN membranes (Fractions 1 and 2; *versus* 15 minutes *P* < 0.0001, *n* = 6), indicating rapid 110kDa TrkA movement from ER to GN, in association with 140kDa TrkA maturation.

The trafficking of labelled TrkAIII differed considerably to that of immature 110kDa (Figure 4B). At 15 minutes, 61.5±5.2% of immature 100kDa TrkAIII was ER-associated (fractions 5 and 6), 28±2.4% COP-associated (fractions 3 and 4) and 10.4±0.9% GN-associated (fractions 1 and 2). By 30 minutes, ER-associated 100kDa TrkAIII had reduced to 26.9+2.3% (fractions 5 and 6; *P* < 0.0001, df = 6), significantly increased to 53.5±4.6% in COP membranes (fractions 3 and 4; *P* < 0.0001, df = 6) and to 19.6±1.8% in GN membranes (fractions 1 and 2; *P* < 0.0001, df = 6). At 45 minutes, 100kDa TrkAIII was 25.7±2.2% ER-associated (fractions 5 and 6; *versus* 15 minutes *P* < 0.0001, df = 6), 39.1±3.3% COP-associated (fractions 3 and 4; significantly increased *versus* 15 minutes *P* < 0.0001, *n* = 6; significantly reduced compared to 30 minutes *P* < 0.0001, df = 6), and 35.2±3.1% GN-associated (fractions 1 and 2; *versus* 30 minutes *P* < 0.0001, df = 6). By 60 minutes 100kDa TrkAIII was 62±5.3% ER-associated (fractions 5 and 6; *versus* 45 minutes *P* < 0.0001, df = 6), 26±2.2% COP-associated (fractions 3 and 4; *versus* 45 minutes *P* < 0.0001, df = 6) and only 12±1% GN-associated (fractions 1 and 2; *versus* 45 minutes; *P* < 0.0001, df = 6), indicating the retrograde transport of TrkAIII back to the ER.

CEP-701 (100nM: 3 hours pre-incubation prior to metabolic labelling, then throughout the time course) (Figure 4C), resulted in a pattern of 100kDa TrkAIII distribution that at 15 minutes was 69.7±6% ER-associated (fractions 5 and 6), 18±1.5% COP-associated (fractions 3 and 4) and 12.3±0.9% GN-associated (fractions 1 and 2), at 30 minutes was significantly reduced to 18.3±2.3% ER-associated (fractions 5 and 6; *P* < 0.0001, df = 6) and significantly increased to 48.9±5.2% COP-associated (fractions 3 and 4;*P* < 0.0001, df = 6) and 32.5±3.5% GN-associated (fractions 1 and 2; *P* < 0.0001, df = 6). Furthermore, a novel 120kDa TrkAIII species was detected in GN membrane fraction 1. Total TrkAIII (100kDa plus 120kDa) was, therefore, 15.8±1.6% ER-associated (fractions 5 and 6), 41.9±3.6% COP-associated (fractions 3 and 4) and 41.3±3.6% GN-associated (fractions 1 and 2). By 60 minutes, CEP-701 had caused a significant reduction in ER-associated TrkAIII to 30.9±3.1% (fractions 5 and 6; *versus* untreated controls; *p* < 0.0001, *n* = 6) and to 23.8±2.4% in COP membranes (fractions 3 and 4; *versus* 30 minutes *P* < 0.0001, df = 6), associated with a significant increase in GN-associated TrkAIII to 45.1±4.5% (fractions 1 and 2; *versus* 30 minutes, *P* = 0.0045, df = 6). This was associated with a marked increase in 120kDa TrkAIII expression, which was distributed 58.7±7% in COP-negative GN membrane fraction 1 and 41.3±4.9% in COP positive GN membrane fraction 2. Total TrkAIII levels (100kDa plus 120kDa) were, therefore, significantly reduced to 22.1±2.9% in ER membranes (fractions 5 and 6; *versus* untreated controls at 60 minutes *P* < 0.0001, df = 6) and to 17.1±2.2% in COP membranes (fractions 3 and 4; *versus* 60 minutes untreated controls; *P* = 0.0012, df = 6), and significantly increased to 60.6±7.8% in GN membranes (fractions 1 and 2; *versus* 60 minutes untreated controls *P* < 0.0001, df = 6). This indicates that CEP-701 promotes TrkAIII trafficking from the ER to GN, resulting in GN-associated 120kDa TrkAIII maturation.

### Spontaneous TrkAIII activation occurs within the ERGIC/COPI vesicle compartment

**Figure 5:**
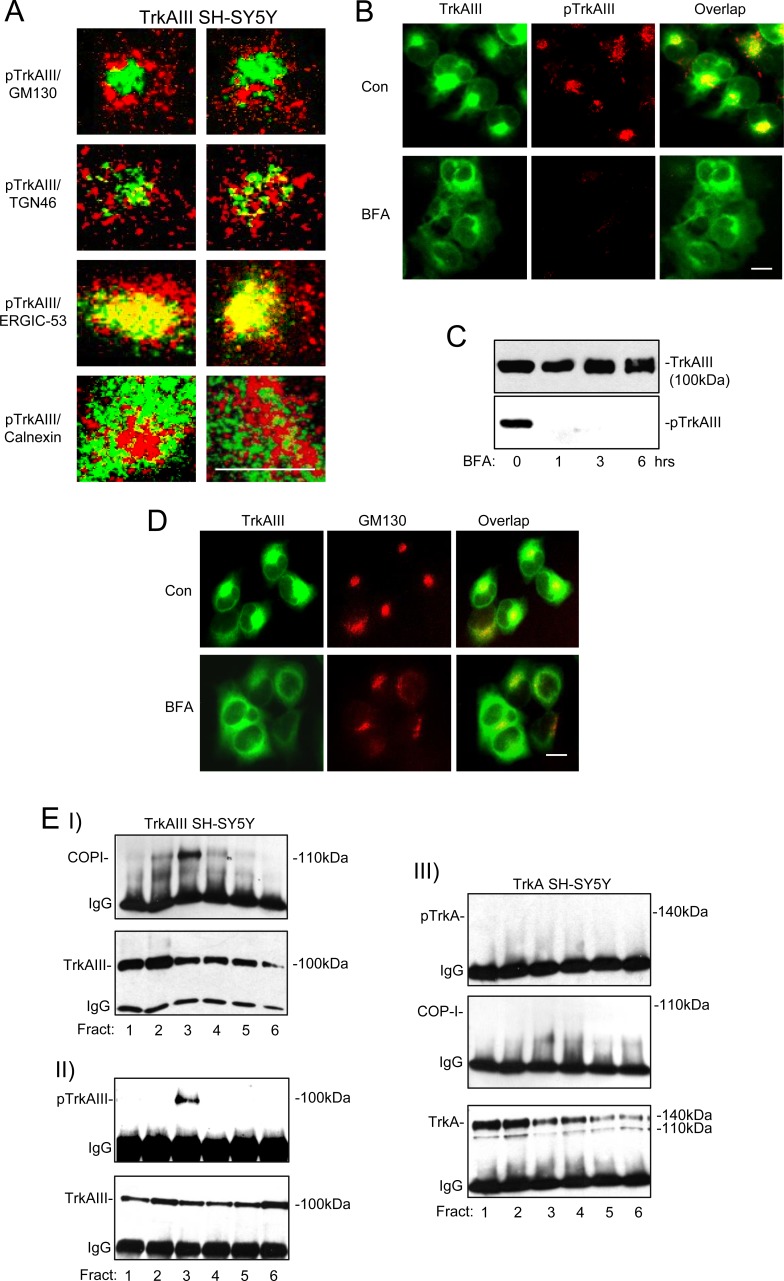
Spontaneous TrkAIII activation occurs within the ERGIC/COPI compartment Representative confocal IF micrographs demonstrating different degrees of association (yellow) between Y490-phosphorylated TrkAIII (pTrkAIII) (red) and GM130 (green) (top panels); pTrkAIII (red) and TGN46 (green) (2^nd^ panels); pTrkAIII (red) and ERGIC-53 (green) (3^rd^ panels); and pTrkAIII (red) and Calnexin (green) (bottom panels) in individual representative TrkAIII SH-SY5Y cells (magnification x100; bar = 10μm). **B.** Indirect non-confocal IF, demonstrating the degree of overlap (yellow) between TrkAIII (green) and phosphorylated TrkAIII (red) in untreated TrkAIII SH-SY5Y cells (Con) (top 3 panels) and BFA-treated (0.1μM for 3 hr) TrkAIII SH-SY5Y cells (bottom 3 panels; bar = 10μm). **C.** Western blots of whole cell extracts (30μg), demonstrating the effect of BFA (0.1μM for 0, 1, 3 and 6 hours) on total and tyrosine phosphorylated TrkAIII. **D.** Indirect non-confocal IF, demonstrating overlap (yellow) and general distribution of TrkAIII (green) and GM130 (red) in untreated (Con) and BFA-treated (0.1μM for 3 hr) TrkAIII SH-SY5Y cells. Western blots demonstrating: **EI.** TrkAIII co-immunoprecipitation of COP-I in density gradient ultracentrifugation-purified TrkAIII SH-SY5Y membranes; **EII.** The presence of tyrosine phosphorylated TrkAIII in TrkAIII immunoprecipitates from the same TrkAIII SH-SY5Y membrane preparation and **EIII.** The absence of TrkA/COPI co-immunoprecipitation and TrkA tyrosine phosphorylated in density gradient ultracentrifugation-purified intracellular membranes from TrkA SH-SY5Y cells.

In TrkAIII SH-SY5Y cells, confocal IF confirmed a close relationship between Y490 phosphorylated TrkAIII and the ERGIC marker ERGIC-53, when compared to the cis-GN marker GM130, the trans-GN marker TGN46 and the ER marker calnexin (Figure 5A), confirming our previous report using non-confocal IF [3].

Brefeldin A (BFA; 5μg/ml for 6 hours) abrogated TrkAIII tyrosine phosphorylation, assessed by both indirect IF (Figure 5B) and Western blotting of whole cell extracts (Figure 5C). BFA caused re-distribution of GM130 in TrkAIII SH-SY5Y cells, consistent with disruption of a constitutively intact GN (Figure 5D).

In co-immunoprecipitation assays, tyrosine phosphorylated TrkAIII pulled down COPI from density-gradient ultracentrifugation purified membrane fraction 3, whereas non-phosphorylated TrkA did not, confirming a different relationship between COP-I, non-phosphorylated TrkA in TrkA SH-SY5Y cells and tyrosine phosphorylated TrkAIII in TrkAIII SH-SY5Y cells, respectively (Figure 5E).

### TrkA inhibitors promote 120kDa TrkAIII maturation

**Figure 6:**
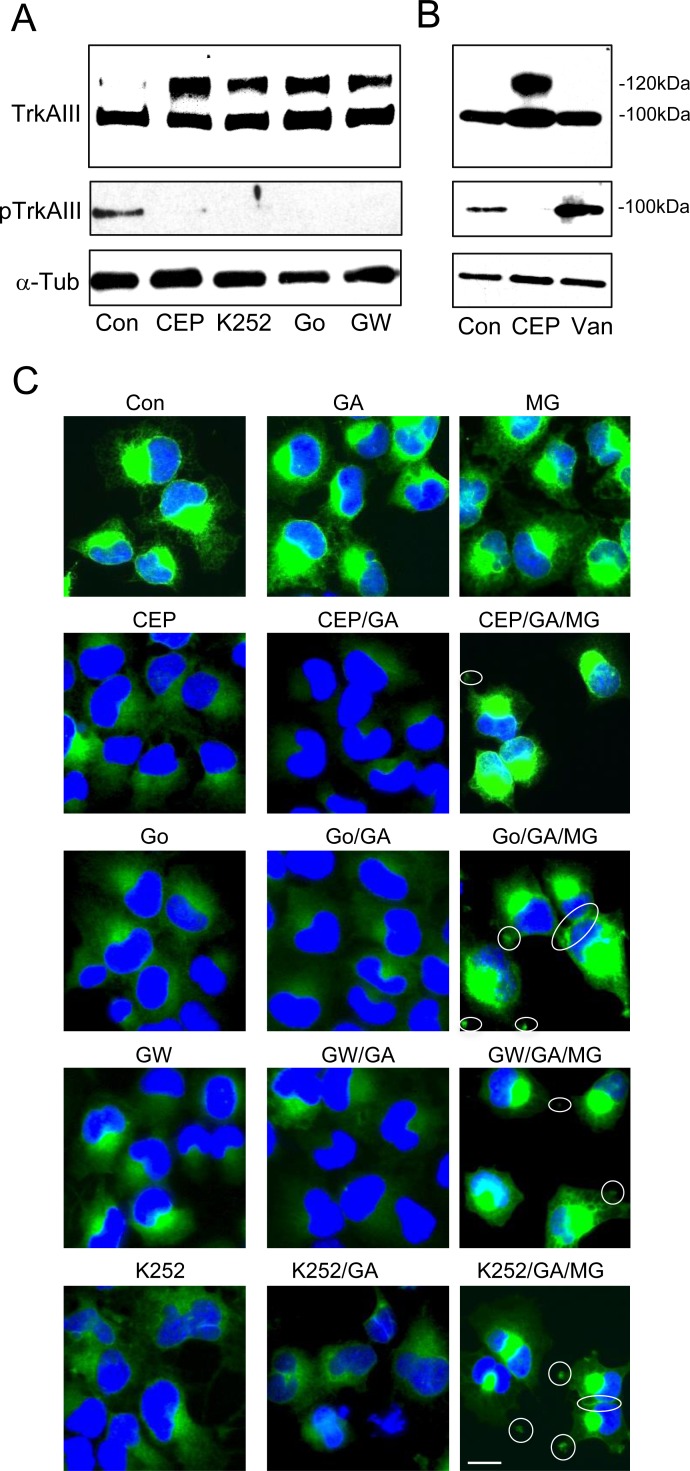
TrkA inhibitors promote 120kDa TrkAIII maturation and degradation **A**. Western blots demonstrating the levels of 100kDa and 120kDa TrkAIII (TrkAIII), tyrosine phosphorylated TrkAIII (pTrkAIII) and α-tubulin in total extracts (30μg) from untreated (con); CEP-701-treated (CEP; 100nM for 6 hours); K252a-treated (K252; 100nM for 6 hours); Go6076-treated (Go; 100nM for 6 hours) and GW441756-treated (GW; 100nM for 6 hours) TrkAIII SH-SY5Y cells. **B.** Western blots comparing the effects of CEP-701 (CEP) and sodium orthovanadate (Van) on 100kDa and 120kDa TrkAIII levels and TrkAIII tyrosine phosphorylation (pTrkAIII). **C.** Indirect IF demonstrating changes in TrkAIII IF intensity and distribution in TrkAIII SH-SY5Y cells treated with CEP-701 (CEP), Go6976 (Go), GW441756 (GW) and K252a (K252), alone or in combination with GA plus or minus MG-132 (MG). Potential sites of cell surface TrkAIII are circled (bar = 10μm).

Different TrkA inhibitors were compared in their capacity to promote 120kDa TrkAIII maturation. The TrkA inhibitors CEP-701, K252a, Go6976 and GW441756 [17-21] all abrogated TrkAIII tyrosine phosphorylation and promoted 120kDa TrkAIII maturation (Figure 6A). In contrast, the hyper-activation of TrkAIII induced by sodium orthovanadate did not result in 120kDa TrkAIII maturation, supporting an inverse relationship between TrkAIII tyrosine phosphorylation and 120kDa maturation (Figure 6B). In IF studies, all TrkA inhibitors decreased TrkAIII staining intensity and promoted more diffuse staining without evidence of cell surface expression, whereas sodium orthovanadate did not alter TrkAIII staining intensity or distribution (Figure 6C). Combination of TrkA inhibitors with the Hsp90 inhibitor Geldanamycin (GA, 1μM) further reduced TrkAIII staining intensity but in the presence of the proteasome inhibitor MG-132 (1μM) TrkAIII staining intensity was restored in association with re-distribution to intercellular junctions and cellular projections, consistent with potential cell surface expression (Figure 6C). These data suggest that TrkA inhibitors and GA promote TrkAIII degradation at the proteasome.

### Nocodozol and GA inhibit 120kDa TrkAIII Maturation

**Figure 7:**
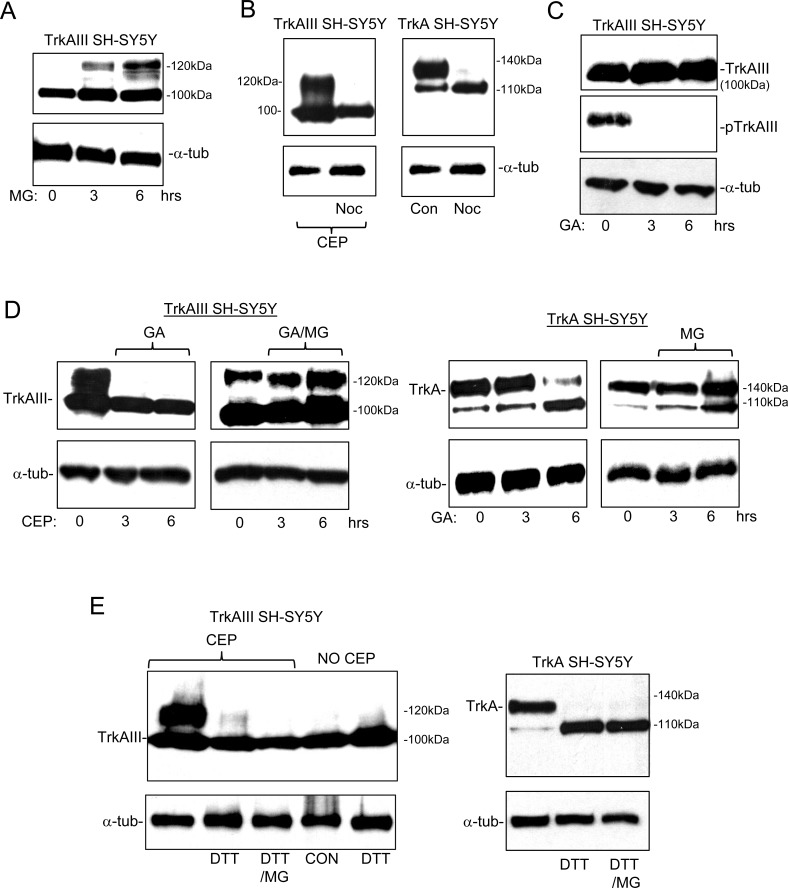
120kDa TrkAIII is degraded at the proteasome, is microtubule-dependent and GA-sensitive Western blots demonstrating: **A.** the effect of MG-132 (1μM for 3 and 6 hours) on 100kDa TrkAIII, 120kDa TrkAIII and α-tubulin (α-tub) levels in TrkAIII SH-SY5Y cells; **B.** the effect of Nocodozol (Noc; 100μM for 6 hours) upon 100kDa TrkAIII, 120kDa TrkAIII and α-tubulin (α-tub) levels in CEP-701-treated (CEP) TrkAIII SH-SY5Y cells (left panel) and upon 110kDa TrkA, 140kDa TrkA and α-tubulin levels in TrkA SH-SY5Y cells (right panel); **C.** The effect of Geldanamycin (GA; 1μM for 3 and 6 hours) on 100kDa TrkAIII, 120kDa TrkAIII, tyrosine phosphorylated TrkAIII (pTrkAIII) and α-tubulin levels in TrkAIII SH-SY5Y cells; **D.** The effect of Geldanamycin (GA; 1μM for 3 and 6 hours) plus or minus MG-132 (MG; 1μM for 3 and 6 hours) on 100kDa TrkAIII, 120kDa TrkAIII and α-tubulin (α-tub) levels in TrkAIII SH-SY5Y cells pre-treated with CEP-701 (CEP; 100nM for 6 hours) to induced 120kDa TrkAIII expression (Left 4 panels), plus the effect of the same combinations of GA plus or minus MG132, in non-CEP-701 treated TrkA SH-SY5Y cells (right 4 panels); **E.** The effect of DTT (100μM for 6 hours) plus or minus MG-132 (MG; 1μM) on 100kDa TrkAIII, 120kDa TrkAIII and α-tubulin levels in CEP-701 pre-treated (CEP; 100nM for 6 hours) and non-CEP-701 treated TrkAIII SH-SY5Y cells (Left panels) and in untreated TrkA SH-SY5Y cells (right panels).

MG-132 (1μM for 3 and 6 hours) induced low-level 120kDa TrkAIII expression, suggesting that in TrkAIII SH-SY5Y cells a proportion of TrkAIII must arrive at the GN but upon 120kDa maturation is degraded at the proteasome and, therefore, not readily detected (Figure 7A).

Nocodozol (0.1μM) abrogated 120kDa TrkAIII maturation induced by CEP-701 in TrkAIII SH-SY5Y cells and also abrogated 140kDa TrkA maturation in TrkA SH-SY5Y cells (Figure 7B), confirming that both 120kDa TrkAIII and 140kDa TrkA maturation is microtubule-dependent, which is consistent with COPI vesicle transport to the GN [22, 23].

GA (1μM for 12 hours) inhibited TrkAIII tyrosine phosphorylation, did not reduce 100kDa TrkAIII levels nor induce 120kDa TrkAIII expression (Figure 7C) but did inhibit 120kDa TrkAIII maturation induced by CEP-701 (Figure 7D). MG-132 not only reversed this GA-inhibitory effect but also augmented 120kDa TrkAIII expression above that induced by CEP-701 alone (Figure 7D), indicating that GA enhances 120kDa TrkAIII degradation at the proteasome. We also confirm our previous report [2], that MG-132 reverses GA abrogation of 140kDa TrkA expression in TrkA SH-SY5Y cells (Figure 7D). GA did not reduce 100kDa TrkAIII or 110kDa TrkA levels, indicating that these immature receptor forms are relatively insensitive to degradation at the proteasome and are not stabilized by GA-sensitive interaction with Hsp90.

The thiol reducing agent dithiothreitol (DTT) inhibits protein folding, promotes protein retention within the ER and prevents protein trafficking to the GN [24]. DTT abrogated both CEP-701-induced 120kDa TrkAIII maturation and constitutive 140kDa TrkA maturation, without reducing immature receptor levels (Figure 7E, left panel). This effect was not inhibited by MG-132, confirming it to be proteasome-independent, consistent with the necessity for transport to the GN for both 120kDa TrkAIII and 140kDa TrkA maturation (Figure 7E).

## DISCUSSION

In this study, we characterise the re-localisation mechanism responsible for spontaneous activation and subsequent oncogenic activity of the TrkAIII oncoprotein in SH-SY5Y NB cells. Exons 6-7 skipping that characterises TrkAIII, eliminates the D4 IG-like domain and several N-glycosylation sites involved in optimising intracellular trafficking and reducing spontaneous activation potential, increasing intracellular accumulation and facilitating spontaneous activation within the ERGIC/COPI vesicle compartment. This, in turn, promotes retrograde TrkAIII transport back to the ER and reduces anterograde transport to the GN, setting up a self-perpetuating mechanism for intracellular TrkAIII entrapment, accumulation, spontaneous activation and oncogenic Akt signalling from this alternative localisation. TrkA tyrosine kinase inhibitors abrogate this mechanism and promote TrkAIII transport to the GN, resulting in GN-associated TrkAIII maturation to a 120kDa species that is more readily degraded at the proteasome.

The marked difference in intracellular trafficking, cell surface expression and spontaneous activation exhibited by TrkA and TrkAIII in SH-SY5Y cells confirms the critical role played exons 6 and 7 encoded sequence in optimising intracellular trafficking from the ER to the GN, reducing intracellular spontaneous activation potential and promoting receptor maturation, an essential step in cell surface expression (this study and [2]). In the case of TrkA, this results in two predominant inactive steady state pools, one within the GN and the other at the cell surface (this study and [1-3]) both maintained inactive by a full complement of spontaneous activation inhibitory D4 and D5 IG-like domains, a full N-glycosylated status that reduces aberrant accumulation within the ERGIC, N-glycan maturation that further reduces aberrant intracellular accumulation by promoting translocation to the cell surface [1-4, 7, 8-10] and, in the case of cell surface TrkA, endogenous PTPase activity [2]. In contrast to TrkA, immature N-glycosylated 100kDa TrkAIII exits the ER but fails to adequately reach the GN. As a consequence, TrkAIII does not undergo significant GN-associated N-glycan maturation, fails to reach the cell surface and becomes entrapped between the ER and ERGIC intracellular membrane compartments, within which it accumulates and undergoes spontaneous ERGIC/COPI-associated activation.

Metabolic labelling comparisons indicated that whilst TrkA and TrkAIII both exit the ER, TrkAIII recycles back to the ER from the ERGIC, resulting in entrapment and accumulation, whereas TrkA moves from the ERGIC to the GN. TrkAIII recycling from the ERGIC to the ER was prevented by TrkA inhibitors, which promoted TrkAIII trafficking to the GN, resulting in GN-associated TrkAIII N-glycan maturation. This indicates that retrograde recycling of TrkAIII back to the ER and reduced transport to the GN is TrkAIII activity-dependent. Subsequent TrkAIII entrapment and accumulation within the pre-GN compartment, combined with omission of the D4 IGC1 spontaneous activation prevention domain, further augment the potential for spontaneous intracellular activation. This novel activation-regulated re-localisation mechanism differs to that utilised by mutated FLT and Kit RTK oncogenes, which fail to satisfy ER quality control and are retained and activated within the ER [10-12], and the Trk-T3 oncogene that is retained and activated within the ERES/COPII compartment as a result of chimeric TFG sequences involved in COPII vesicle formation [13].

TrkAIII activation within an ERGIC/COPI vesicle context was confirmed using BFA, an inhibitor of ARF-1-dependent COPI vesicle formation [21], which abrogated ERGIC-associated TrkAIII activation, was supported by the close relationship exhibited between COP-I positive membranes and tyrosine phosphorylated TrkAIII and by co-immunoprecipitation of tyrosine phosphorylated TrkAIII and COP-I from the same membrane fraction. The fact that BFA inhibited TrkAIII activity but did not promote TrkAIII 120kDa maturation is explained by its capacity to disrupt the GN (this study and [21]), which would prevent the transport of inactivated TrkAIII to the GN. BFA does not disrupt the ERGIC [25], further supporting the hypothesis of ERGIC-associated TrkAIII activation within a COP-I vesicle context but does prevent COP-II vesicle formation [21]. We do not exclude, therefore, a potential role for COP-II vesicles in regulating the spontaneous activation of TrkAIII. BFA inhibition of spontaneous TrkAIII activation in SH-SY5Y cells differs to a report that ERGIC-associated spontaneous TrkA activation in human kidney 293 cells is insensitivity to BFA and, therefore, COPI-independent [14].

The mechanism through which activated TrkAIII recycles back to the ER remains to be elucidated. TrkAIII does not contain KDEL-like sequences, suggesting a KDEL-R-independent mechanism but could complex with KDEL-cargos in active-form to facilitate retrograde transport in KDEL-R/COPI vesicles [26, 27]. KDEL-R is a G-protein coupled receptor (GPCR) and induces Arf1-dependent COPI vesicle formation upon cargo binding [27]. GPRCs activate immature intracellular TrkA [28-30], suggesting an intriguing possibility that cargo-activated KDEL-Rs may activate TrkAIII, resulting in retrograde transport of KDEL-R/COPI vesicles containing KDELR-activated TrkAIII. Although immature TrkA receptors were not activated under similar conditions, this may explain the selective activation of TrkAIII within the ERGIC/COPI compartment. We are investigating this possibility.

Alternatively, the recycling of activated TrkAIII from the ERGIC to ER may result from motor protein-driven, MT minus-end, retrograde receptor transport, analogous to that described for the retrograde transport of ligand-activated cell surface TrkA receptors [31-33]. Such a mechanism would not only explain recycling back to the ER but also the accumulation of active TrkAIII at the centrosome [3]. Whatever the mechanism, the net result is self-perpetuating TrkAIII re-localisation to the ER/ERGIC compartment, with the subsequent combination of entrapment and accumulation of this compromised receptor augmenting the potential for spontaneous activation.

TrkAIII activation within ERGIC/COPI compartment differs to activation of the TrkT3 oncogene, which occurs within the ERES/COPII compartment [13]. This can be explained by different re-localisation mechanisms. TrkT3 is trapped within the ERES/COPII compartment by chimeric TFG sequences, not present in TrkAIII, which regulate COPII vesicle formation [13]. Both mechanism, however, result in spontaneous activation and oncogenic Akt signalling from their respective locations, confirming that TrkA oncoproteins, compromised either by mutation or alternative splicing, may re-localise to different intracellular membranes but become activated when they accumulate above the local threshold for spontaneous activation.

In contrast to TrkAIII, TrkA did not exhibit spontaneous activation in SH-SY5Y cells, clearly implicating the extracellular D4 IG-like domain and associated N-glycosylation sites omitted from TrkAIII, in preventing spontaneous intracellular TrkA activation. This most likely depends upon the optimisation of intracellular TrkA trafficking to the GN, GN-associated N-glycan maturation and subsequent expression at the cell surface [7-9]. The optimisation of intracellular TrkA trafficking to the GN appears to be key in this respect, as illustrated by the recent report of spontaneous ERGIC-associated activation of immature TrkA in human kidney 293 cells [14]. This cell specific effect, characterised by aberrant TrkA accumulation within the ERGIC above the spontaneous activation threshold, results in GN disruption, which promotes the entrapment of immature TrkA within the ERGIC, illustrating the absolute requirement for optimised intracellular trafficking to the GN in preventing spontaneous intracellular activation [14].

TrkAIII did not exhibit spontaneous activation throughout the ER but could be activated throughout the ER by the PTPase inhibitor sodium orthovanadate, implicating ER PTPases in maintaining ER-associated TrkAIII inactive [2]. This indicates that active TrkAIII recycling back to the ER would be inactivated within the ER prior to being re-transport back to the ERGIC in COPII vesicles, the formation of which would be enhanced by TrkAIII-induced Akt signalling [1, 26]. Spontaneous TrkAIII activation within the GN was also minimal. This may reflect either the low level of TrkAIII in COP-negative GN membranes and/or the RTK inhibitory nature of the GN, which is exemplified by the steady state GN-associated pool of inactive TrkA, consistent with the GN function as natural reservoir for receptor tyrosine kinases during their maturation [34].

TrkA inhibitors promoted TrkAIII transport from the ER to GN, resulting in GN-associated TrkAIII maturation to 120kDa, analogous to GN-associated TrkA maturation from 110kDa to 140kDa (this study and [1, 3]). In contrast, hyper-phosphorylation of TrkAIII induced by sodium orthovanadate did not result in 120kDa TrkAIII maturation, supporting an inverse relationship between TrkAIII activation and maturation. This bears similarity to ERGIC-associated activation of immature 110kDa TrkA receptors in human kidney 293 cells, which impedes 140kDa TrkA maturation by disrupting the GN [14]. Here, we found no evidence for GN disruption in TrkAIII SH-SY5Y cells, suggesting that impaired maturation of TrkAIII involves the reduced transport of activated TrkAIII to the GN, rather than GN disruption.

The proteasome inhibitor MG132 promoted low-level expression of 120kDa TrkAIII without influencing 100kDa TrkAIII levels in TrkAIII SH-SY5Y cells. This indicates that a small proportion of TrkAIII must constitutively arrive at the GN and mature to 120kDa but is not detected in the absence of MG132 due to degradation at the proteasome. Furthermore, MG132 augmented 120kDa TrkAIII levels induced by CEP-701, restored 120kDa TrkAIII expression to CEP-701-treated TrkAIII SH-SY5Y cells in which 120kDa TrkAIII expression had been abrogated by the Hsp90 inhibitor GA and, in the presence of TrkA inhibitors, caused the re-distribution of TrkAIII to cellular projections and intracellular junctions, suggesting cell surface expression. Together, these data indicate that: TrkA inhibitors promote GN-associated TrkAIII 120kDa maturation; 120kDa TrkAIII is more sensitive than 100kda TrkAIII to degradation at the proteasome; 120kda TrkAIII is stabilised by GA sensitive interaction the Hsp90 adding to our report that TrkAIII exhibits GA-sensitive interaction with Hsp90 [2], and that cell surface expression of 120kDa TrkAIII is prevented by its degradation at the proteasome.

In conclusion, we propose that the novel re-localisation mechanism characterised by spontaneous ERGIC/COPI-associated activation and subsequent retrograde recycling back to the ER is responsible for the entrapment, accumulation, spontaneous activation and subsequent oncogenic activity of TrkAIII and is initiated from the alternative location of the ERGIC/COPI compartment. This highlights the importance of intracellular TrkAIII re-localisation for oncogenic activity, identifies the ERGIC/COPI compartment as a preferential site for spontaneous TrkAIII activation and provides an explanation for how TrkAIII, compromised by the omission of the D4 IGC1 spontaneous activation-prevention domain, is able to overcome the threshold for spontaneous activation. The subsequent and altered oncogenic signalling through PI3K/Akt but not Ras/MAPK results in increased survival, a pattern of malignant gene expression, increased genetic instability and promotion of stem cell-like behaviour rather than differentiation [1, 3, 4, 6, 35], which together not only promote tumour progression but may also be involved in tumour initiation. TrkA tyrosine kinase inhibitors abrogate this mechanism by promoting the anterograde transport of inactivated TrkAIII to the GN, resulting in GN-associated TrkAIII maturation to a 120kDa form that is degraded at the proteasome, reducing both intracellular accumulation and oncogenic activity of the TrkAIII oncoprotein.

## MATERIALS AND METHODS

### Cell lines and reagents

PcDNA SH-SY5Y, TrkA SH-SY5Y, and TrkAIII SH-SY5Y cell lines were grown in recommended medium (RPMI or Dulbecco's modified Eagle's medium) supplemented with 10% foetal calf serum and appropriate antibiotics (phleomycin [Zeocin], penicillin, and streptomycin), as described previously [1-4]. Cell culture reagents, MG-132, geldanamycin, cytochalasin D, nocodozol, proteinase inhibitor Cocktail, Bis-benzimide tri-hydrochloride, propidium iodide, Histodenz^TM^, NGF, K252a, Go6976 and GW441756 were purchased from Sigma-Aldrich (St. Louis MO). Endoglycosidase H was from Roche Italia (Milan, It). The pan Trk inhibitor CEP-701 was from Cephalon Inc. (West Chester, PA). Polyclonal anti-TrkA (C14) and monoclonal anti-Phospho-tyrosine (pY99) antibodies were from SantaCruz (Santa Cruz, Ca). The polyclonal anti-α-tubulin antibody was from Sigma (St. Louis, MO). Polyclonal anti-phospho-Y674/675 TrkA, anti-COPI, COPII and mouse monoclonal anti-Calnexin and anti-TGN46 antibodies were from Abcam (Cambridge, UK). The polyclonal anti-phospho-Y490 TrkA antibody was from Cell signalling (Danvers, MA). Mouse monoclonal anti-GM130 antibody was from BD Bioscience (San Jose, Ca). FITC and TRIC-conjugated secondary anti mouse and anti-rabbit IgG antibodies were from Jackson Immune Research (Bar Harbor, Maine). VectorMount^TM^ medium was from Vector Laboratories (Berlingame, Ca).

### Histodenz^TM^ ultracentrifugation fractionation of internal cell membranes

Briefly, cells were incubated with cytochalasin D (1μg/ml) and nocodozol (0.1μM) for 1 hour at 37°C prior to harvest, scraped into cold PBS, washed in homogenisation buffer (130mM KCl, 5mM MgCl_2_, 25mM Tris-HCl pH 7.3) and homogenised in 500μl homogenisation buffer containing proteinase inhibitor cocktail (1mM 4-(−2 amino ethyl) benzene-sulfanyl-fluoride hydrochloride, 20μM leupeptin, 15μM pepstatin and 15μM chymostatin). Homogenates were centrifuged for 5 minutes at 1000xg at 4°C, supernatants were collected and re-centrifuged at 1000xg for 5 minutes at 4°C to give the post nuclear supernatant (PNS). The PNS was adjusted to 500μl in homogenisation buffer and layered on top of a step-gradient from bottom to top, of 500μl of 40%, 1ml of 20%; 1ml of 17.5% and 1ml of 15% Histodenz^TM^ (Sigma-Aldrich) in homogenisation buffer. Gradients were ultra centrifuged at 4°C in a Sw55Ti rotor for 60 minutes at 100,000xg and 500μl fractions collected (top to bottom) and mixed with 125μl of 5x detergent buffer (5%NP-40, 2.5% deoxycholate, 0.5% SDS, 250mM Tris-HCl pH 8.0). Fractions were analysed directly by Western blot or subjected to immunoprecipitation/Western blot. Immunoprecipitates were washed with RIPA buffer (1% NP-40, 0.5% deoxycholate, 0.1% SDS, 150mM NaCl and 50mM Tris-HCl pH 8.0); twice with high salt buffer (0.2% SDS, 1% TritonX-100, 5mM EDTA, 500mM NaCl and 50mM Tris-HCl pH 8.0) and once with TEN (50mM Tris-HCl pH 8.0; 5mM EDTA and 150mM NaCl), prior to being subjected to reducing SDS PAGE/Western blotting.

### Immunoprecipitation and western blots

Cells were extracted in lysis buffer (PBS containing 0.5% sodium deoxycholate, 1% NP40, 0.1% SDS, 1mM sodium orthovanadate, 1mM PMSF, 1μg/ml of pepstatin A and Aprotinin), protein concentrations were calculated by Bradford Assay (Sigma-Aldrich) and extracts pre-cleared with 1μg pre-immune IgG (1 hour at 4°C) and 20μl protein A Sepharose (Fast Flow, Sigma) for 20 minutes at 4°C. For immunoprecipitation, pre-cleared extracts (200-500μg for stable transfectants) were incubated with primary antibody at a concentration of 0.1-1μg/500μg total protein for 2-16 hours at 4°C. Following incubation, 20μl Protein A Sepharose (Fast flow, Sigma-Aldrich) in lysis buffer, was added and reactions incubated for 60 minutes at 4°C. Protein sepharose/IgG conjugates were collected by centrifugation (10,000xg for 5 minutes), washed 3 times in lysis buffer, re-suspended in SDS-PAGE sample buffer and subjected to reducing SDS-PAGE/Western blotting. Proteins were trans-blotted by electrophoresis onto Hybond C+ nitrocellulose membranes (Amersham Int. UK) and membranes air-dried. Non-specific protein binding site were blocked by 2 hours incubation in 5% non-fat milk in TBS, prior to incubation for 2-16 hours with primary antibodies diluted in blocking solution at 4°C. Following incubation, membranes were washed in TBS, incubated for 20 minutes with secondary HRP-conjugated antibody diluted in blocking solution and immunoreactive species detected by chemiluminescence reaction, as directed (Amersham Int., Bedford, UK).

### Metabolic labelling of TrkAI and TrkAIII

Briefly, 3-4 million cells per time point, pre-incubated for 8 hours in the presence or absence of 100nM CEP-701, were incubated for 2 hour in methionine and cysteine-free medium prior to exposure for 30 minutes to 100μCi/ml S^35^-labelled methionine/cysteine (Perkin-Elmer). Cells were then washed with pre-warmed complete medium and chased in excess (1mM) cold methionine/cysteine (Sigma-Aldrich), for different times. Cell aliquots were taken at each time point and subjected to Histodenz^TM^ ultracentrifugation fractionation as described above, membrane fractions separated by regular reducing SDS-PAGE and dried gels were subjected to autoradiography. Densitometric analysis was performed using Image J software.

### Regular and confocal immunofluorescence (IF)

Cells grown on Nunc glass chamber slides (Sigma-Aldrich) were washed in PBS, fixed in 37% formaldehyde, permeabilized in cold methanol and processed for indirect immunofluorescence (IF). Slides were incubated for 1 h in blocking solution (1% bovine serum albumin in PBS-0.03% TX100) and then for 2 to 16 h with primary antibody in blocking solution at room temperature. Slides were washed three times in PBS-0.03% TX100, then incubated with secondary fluorochrome-conjugated antibody diluted in blocking solution for 1 h at room temperature, and then washed in PBS-0.03% TX100 and mounted using Vector Mount. For confocal analysis, slides were examined under a Nikon Eclipse 80i confocal microscope and images elaborated using EZ-C1 Gold software (version 3.80). For non-confocal analysis, slides were examined under a Zeiss Axioplan 2 fluorescence microscope, with digital camera and Leica M500 Image Manager software.

### Statistical analysis

Data were analysed statistically by Student's t-test. Statistical significance was associated with probabilities of < 0.05.
